# (1*R*,4*R*,6*S*,7*R*)-5,5-Di­bromo-1,4,8,8-tetra­methyl­tri­cyclo­[5.4.1.0^4,6^]dodecan-12-one

**DOI:** 10.1107/S1600536814007351

**Published:** 2014-04-09

**Authors:** Mohamed Zaki, Ahmed Benharref, Jean-Claude Daran, Moha Berraho

**Affiliations:** a Laboratoire de Chimie des Substances Naturelles, "Unité Associé au CNRST (URAC16)", Faculté des Sciences Semlalia, BP 2390 Bd My Abdellah, 40000 Marrakech, Morocco; b Laboratoire de Chimie de Coordination, 205 Route de Narbone, 31077 Toulouse Cedex 04, France

## Abstract

The title compound, C_16_H_24_Br_2_O, was synthesized from the reaction of β-himachalene (3,5,5,9-tetra­methyl-2,4a,5,6,7,8-hexa­hydro-1*H*-benzo­cyclo­heptene), which was isolated from Atlas cedar (*Cedrus atlantica*). The asymmetric unit contains two independent mol­ecules with similar conformations. Each mol­ecule is built up from two fused seven-membered rings and an additional three-membered ring. In both mol­ecules, one of the seven-membered rings has a chair conformation, whereas the other displays a screw-boat conformation.

## Related literature   

For background to β-himachalene, see: El Haib *et al.* (2011 ▶). For the reactivity of this sesquiterpene and its derivatives, see: El Jamili *et al.* (2002 ▶); Benharref *et al.* (2013 ▶); Oukhrib *et al.* (2013 ▶). For their potential anti­fungal activity against the phytopathogen *Botrytis cinerea*, see: Daoubi *et al.* (2004 ▶). For puckering parameters, see: Cremer & Pople (1975 ▶). 
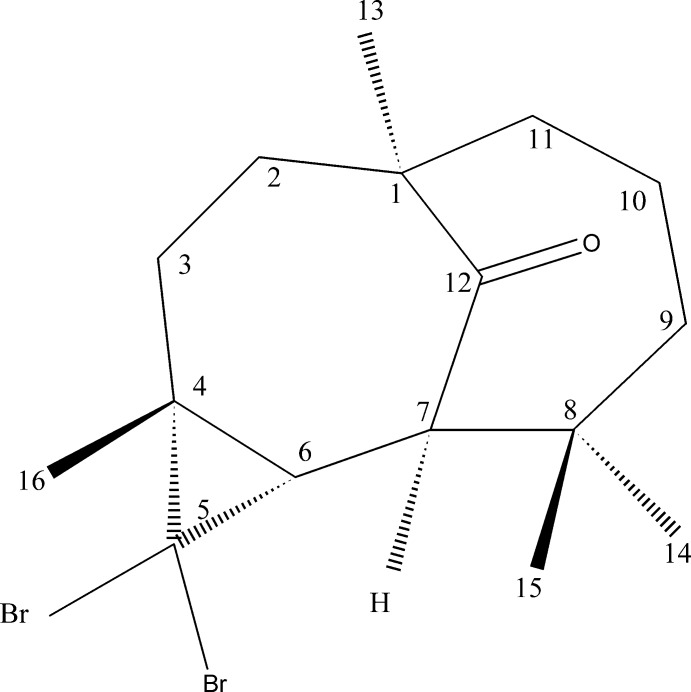


## Experimental   

### 

#### Crystal data  

C_16_H_24_Br_2_O
*M* *_r_* = 392.17
Triclinic, 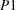

*a* = 6.6550 (3) Å
*b* = 9.4142 (4) Å
*c* = 12.9389 (13) Å
α = 86.008 (6)°
β = 83.921 (6)°
γ = 89.511 (4)°
*V* = 804.13 (9) Å^3^
*Z* = 2
Mo *K*α radiation
μ = 5.03 mm^−1^
*T* = 173 K
0.38 × 0.11 × 0.10 mm

#### Data collection  

Agilent Xcalibur (Eos, Gemini ultra) diffractometer
Absorption correction: multi-scan (*CrysAlis PRO*; Agilent, 2012 ▶) *T*_min_ = 0.670, *T*_max_ = 1.00
11451 measured reflections
6327 independent reflections
5209 reflections with *I* > 2σ(*I*)
*R*_int_ = 0.057

#### Refinement  

*R*[*F* ^2^ > 2σ(*F* ^2^)] = 0.054
*wR*(*F* ^2^) = 0.119
*S* = 1.01
6327 reflections
351 parameters
3 restraints
H-atom parameters constrained
Δρ_max_ = 0.77 e Å^−3^
Δρ_min_ = −0.62 e Å^−3^
Absolute structure: Flack & Bernardinelli (2000 ▶), 3035 Friedel pairs
Absolute structure parameter: −0.017 (15)

### 

Data collection: *CrysAlis PRO* (Agilent, 2012 ▶); cell refinement: *CrysAlis PRO*; data reduction: *CrysAlis PRO*; program(s) used to solve structure: *SHELXS97* (Sheldrick, 2008 ▶); program(s) used to refine structure: *SHELXL97* (Sheldrick, 2008 ▶); molecular graphics: *ORTEP-3 for Windows* (Farrugia, 2012 ▶); software used to prepare material for publication: *publCIF* (Westrip, 2010 ▶).

## Supplementary Material

Crystal structure: contains datablock(s) I. DOI: 10.1107/S1600536814007351/bt6972sup1.cif

Structure factors: contains datablock(s) I. DOI: 10.1107/S1600536814007351/bt6972Isup2.hkl

Supporting information file. DOI: 10.1107/S1600536814007351/bt6972Isup3.cml

CCDC reference: 995040

Additional supporting information:  crystallographic information; 3D view; checkCIF report

## Figures and Tables

**Fig. 1.**
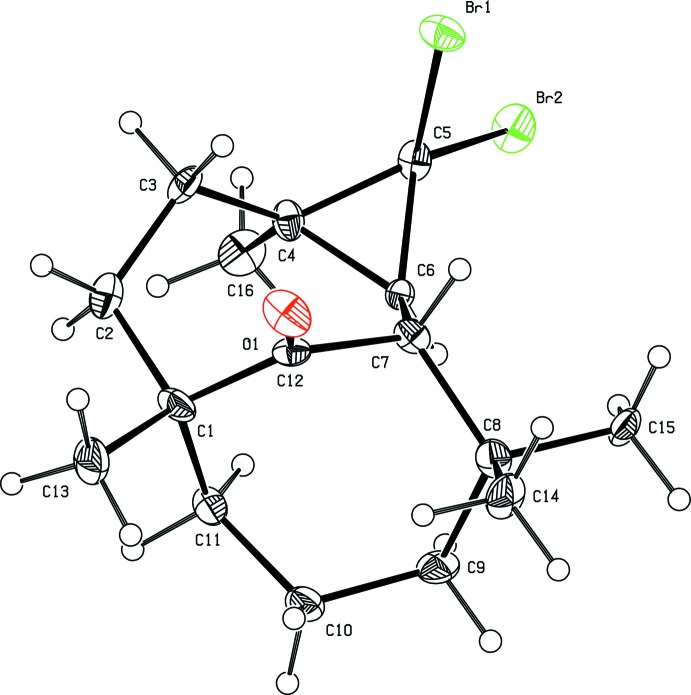
: Molecular structure of the title compound with the atom-labelling scheme. Displacement ellipsoids are drawn at the 30% probability. level. H atoms are represented as small spheres of arbitrary radii.

## supplementary crystallographic information

### 1. Comment

Our work lies within the framework of the valorization of the most abundant essential oils in Morocco, such as the one from Cedrus atlantica. This oil is made up mainly (75%) of bicyclic sesquiterpenes hydrocarbons, among which is found β-himachalene (El Haib *et al.*, 2011). The reactivity of this sesquiterpene and its derivatives has been studied extensively by our team in order to prepare new products having biological properties (El Jamili *et al.*, 2002; Benharref *et al.*, 2013; Oukhrib *et al.*, 2013). Indeed, these compounds were tested, using the food poisoning technique, for their potential antifungal activity against phytopathogen Botrytis cinerea (Daoubi *et al.*, 2004). In this work we present the crystal structure of the title compound. The asymmetric unit contains two independent molecules with almost identical conformations (Fig. 1). Each molecule is built up from two fused seven-membered rings, one having a chair conformation as indicated by the total puckering amplitude QT = 0.8469 (8) Å and spherical polar angle θ = 38.29 (6)° with φ2 = 126.14 (8)°, and φ3 = -139.18 (6)°, while the other shows a screw boat conformation, with QT = 1.0407 (8) Å, θ = 76.80 (4)°, φ2 = 153.32 (4)° and φ3 = 115.21 (2)° (Cremer & Pople, 1975). Owing to the presence of Br atoms, the absolute configuration could be fully confirmed, by refining the Flack parameter (Flack & Bernardinelli, 2000) as C1(*R*), C4(*R*), C6(*S*) and C7(*R*).

### 2. Experimental

To obtain the title compound, BF_3_—Et_2_O(1 mL) was added dropwise to a solution of (1*S*,2*R*,7*R*,8*S*,10*R*)-9,9-dibromo- 1α,2α-epoxy-2,6,6,10-tetramethyltricyclo[5.5.0.0^8^,^10^]dodecane (1 g, 2.5 mmol) in 60 ml of dichloromethane at 195 K under nitrogen. The reaction mixture was stirred for two hours at a constant temperature of 195 K and left at ambient temperature for 24 h. Water (60 ml) was added in order to separate the two phases, and the organic phase was dried and concentrated. The residue obtained was chromatographed on silica-gel eluting with hexane-ethyl acetate (98/2), which allowed the isolation of pure(1*S*,6*R*,7*S*,9*R*)-12-acetyl-8,8-dibromo-5,5,9- trimethyltricyclo[4.4.0,1_7_,_9_]decane in a yield of 20% (196 mg, 0.5 mmol). The title compound was recrystallized from its pentane solution.

### 3. Refinement

All H atoms were fixed geometrically and treated as riding with C—H = 0.96 Å (methyl), 0.97 Å (methylene), 0.98 Å (methine) with *U*_iso_(H) = 1.2*U*_eq_(methylene, methine) or *U*_iso_(H) = 1.5*U*_eq_(methyl). The methyl groups were allowed to rotate but not to tip.

### Crystal data

**Table:**

C_16_H_24_Br_2_O	*Z* = 2
*M**_r_* = 392.17	*F*(000) = 396
Triclinic, *P*1	*D*_x_ = 1.620 Mg m^−^^3^
Hall symbol: P 1	Mo *K*α radiation, λ = 0.71073 Å
*a* = 6.6550 (3) Å	Cell parameters from 2828 reflections
*b* = 9.4142 (4) Å	θ = 3.7–26.6°
*c* = 12.9389 (13) Å	µ = 5.03 mm^−^^1^
α = 86.008 (6)°	*T* = 173 K
β = 83.921 (6)°	Needle, colourless
γ = 89.511 (4)°	0.38 × 0.11 × 0.10 mm
*V* = 804.13 (9) Å^3^	

### Data collection

**Table:**

Agilent Xcalibur (Eos, Gemini ultra) diffractometer	6327 independent reflections
Radiation source: fine-focus sealed tube	5209 reflections with *I* > 2σ(*I*)
Graphite monochromator	*R*_int_ = 0.057
Detector resolution: 16.1978 pixels mm^-1^	θ_max_ = 26.4°, θ_min_ = 3.2°
ω scans	*h* = −8→8
Absorption correction: multi-scan (*CrysAlis PRO*; Agilent, 2012)	*k* = −11→11
*T*_min_ = 0.670, *T*_max_ = 1.00	*l* = −16→16
11451 measured reflections	

### Refinement

**Table:**

Refinement on *F*^2^	Secondary atom site location: difference Fourier map
Least-squares matrix: full	Hydrogen site location: inferred from neighbouring sites
*R*[*F*^2^ > 2σ(*F*^2^)] = 0.054	H-atom parameters constrained
*wR*(*F*^2^) = 0.119	*w* = 1/[σ^2^(*F*_o_^2^) + (0.0447*P*)^2^] where *P* = (*F*_o_^2^ + 2*F*_c_^2^)/3
*S* = 1.01	(Δ/σ)_max_ < 0.001
6327 reflections	Δρ_max_ = 0.77 e Å^−^^3^
351 parameters	Δρ_min_ = −0.62 e Å^−^^3^
3 restraints	Absolute structure: Flack & Bernardinelli (2000), 3035 Friedel pairs
Primary atom site location: structure-invariant direct methods	Absolute structure parameter: −0.017 (15)

### Special details

**Table:**

Geometry. All e.s.d.'s (except the e.s.d. in the dihedral angle between two l.s. planes) are estimated using the full covariance matrix. The cell e.s.d.'s are taken into account individually in the estimation of e.s.d.'s in distances, angles and torsion angles; correlations between e.s.d.'s in cell parameters are only used when they are defined by crystal symmetry. An approximate (isotropic) treatment of cell e.s.d.'s is used for estimating e.s.d.'s involving l.s. planes.
Refinement. Refinement of *F*^2^ against ALL reflections. The weighted *R*-factor *wR* and goodness of fit *S* are based on *F*^2^, conventional *R*-factors *R* are based on *F*, with *F* set to zero for negative *F*^2^. The threshold expression of *F*^2^ > σ(*F*^2^) is used only for calculating *R*-factors(gt) *etc*. and is not relevant to the choice of reflections for refinement. *R*-factors based on *F*^2^ are statistically about twice as large as those based on *F*, and *R*- factors based on ALL data will be even larger.

### Fractional atomic coordinates and isotropic or equivalent isotropic displacement parameters (Å^2^)

**Table:**

	*x*	*y*	*z*	*U*_iso_*/*U*_eq_	
C1	0.5880 (12)	−0.1550 (8)	1.2375 (6)	0.0276 (18)	
C2	0.5505 (13)	−0.0914 (9)	1.3464 (6)	0.034 (2)	
H2A	0.4417	−0.1471	1.3886	0.040*	
H2B	0.6748	−0.1053	1.3818	0.040*	
C3	0.4926 (12)	0.0666 (9)	1.3462 (7)	0.028 (2)	
H3A	0.4717	0.0941	1.4191	0.034*	
H3B	0.6059	0.1241	1.3098	0.034*	
C4	0.3007 (11)	0.1013 (8)	1.2934 (6)	0.0252 (18)	
C5	0.2942 (11)	0.2374 (8)	1.2238 (6)	0.0225 (17)	
C6	0.3148 (11)	0.0978 (7)	1.1734 (6)	0.0191 (17)	
H6	0.1873	0.0660	1.1475	0.023*	
C7	0.5057 (11)	0.0591 (8)	1.1050 (6)	0.0226 (17)	
H7	0.5764	0.1510	1.0817	0.027*	
C8	0.4504 (11)	−0.0078 (8)	1.0021 (6)	0.0275 (18)	
C9	0.3174 (14)	−0.1435 (8)	1.0270 (7)	0.033 (2)	
H9A	0.1841	−0.1137	1.0599	0.039*	
H9B	0.2948	−0.1823	0.9600	0.039*	
C10	0.3944 (13)	−0.2635 (8)	1.0963 (7)	0.032 (2)	
H10A	0.5336	−0.2882	1.0683	0.039*	
H10B	0.3086	−0.3484	1.0942	0.039*	
C11	0.3955 (12)	−0.2268 (8)	1.2106 (6)	0.0281 (19)	
H11A	0.2794	−0.1633	1.2280	0.034*	
H11B	0.3738	−0.3158	1.2557	0.034*	
C12	0.6585 (11)	−0.0347 (8)	1.1572 (6)	0.0221 (17)	
C13	0.7592 (14)	−0.2668 (10)	1.2418 (8)	0.039 (2)	
H13A	0.7752	−0.3149	1.1767	0.059*	
H13B	0.7249	−0.3370	1.3004	0.059*	
H13C	0.8859	−0.2193	1.2510	0.059*	
C14	0.6448 (14)	−0.0429 (10)	0.9350 (6)	0.037 (2)	
H14A	0.6112	−0.0806	0.8703	0.056*	
H14B	0.7220	−0.1143	0.9732	0.056*	
H14C	0.7259	0.0437	0.9186	0.056*	
C15	0.3312 (14)	0.1068 (9)	0.9411 (6)	0.031 (2)	
H15A	0.3025	0.0710	0.8748	0.046*	
H15B	0.4121	0.1939	0.9274	0.046*	
H15C	0.2038	0.1277	0.9825	0.046*	
C16	0.1080 (12)	0.0472 (9)	1.3566 (7)	0.037 (2)	
H16A	0.0519	0.1213	1.4010	0.055*	
H16B	0.1382	−0.0379	1.4004	0.055*	
H16C	0.0094	0.0233	1.3094	0.055*	
O1	0.8370 (8)	−0.0058 (6)	1.1385 (5)	0.0370 (15)	
Br1	0.51009 (11)	0.37150 (8)	1.21049 (7)	0.0329 (3)	
Br2	0.04085 (11)	0.33708 (9)	1.21682 (7)	0.0376 (3)	
C1A	0.2236 (11)	0.6190 (8)	0.6230 (6)	0.0250 (17)	
C2A	0.2439 (12)	0.5548 (8)	0.5146 (6)	0.0284 (19)	
H2C	0.1584	0.6115	0.4690	0.034*	
H2D	0.3858	0.5664	0.4836	0.034*	
C3A	0.1869 (11)	0.3985 (9)	0.5135 (7)	0.024 (2)	
H3C	0.2845	0.3398	0.5505	0.029*	
H3D	0.1994	0.3720	0.4403	0.029*	
C4A	−0.0271 (11)	0.3624 (8)	0.5636 (6)	0.0237 (17)	
C5A	−0.0676 (11)	0.2274 (8)	0.6295 (6)	0.0208 (17)	
C6A	−0.0769 (11)	0.3672 (7)	0.6818 (6)	0.0193 (16)	
H6A1	−0.2177	0.3998	0.7029	0.023*	
C7A	0.0724 (11)	0.4048 (7)	0.7548 (6)	0.0208 (16)	
H7A1	0.1292	0.3125	0.7819	0.025*	
C8A	−0.0317 (11)	0.4772 (7)	0.8529 (5)	0.0233 (17)	
C9A	−0.1548 (13)	0.6092 (8)	0.8239 (6)	0.027 (2)	
H9A1	−0.2120	0.6492	0.8895	0.032*	
H9A2	−0.2699	0.5776	0.7887	0.032*	
C10A	−0.0473 (13)	0.7314 (8)	0.7539 (6)	0.034 (2)	
H10C	−0.1388	0.8148	0.7518	0.040*	
H10D	0.0745	0.7597	0.7852	0.040*	
C11A	0.0146 (11)	0.6919 (8)	0.6438 (6)	0.0258 (18)	
H11C	0.0137	0.7796	0.5969	0.031*	
H11D	−0.0896	0.6275	0.6240	0.031*	
C12A	0.2523 (11)	0.4925 (7)	0.7042 (6)	0.0237 (17)	
C13A	0.3950 (13)	0.7266 (9)	0.6268 (7)	0.035 (2)	
H13D	0.3753	0.8098	0.5791	0.053*	
H13E	0.5252	0.6818	0.6059	0.053*	
H13F	0.3939	0.7566	0.6979	0.053*	
C14A	−0.1763 (13)	0.3701 (8)	0.9151 (7)	0.032 (2)	
H14D	−0.2433	0.4139	0.9761	0.049*	
H14E	−0.1004	0.2861	0.9378	0.049*	
H14F	−0.2782	0.3416	0.8711	0.049*	
C15A	0.1302 (13)	0.5154 (8)	0.9242 (6)	0.0308 (19)	
H15D	0.2236	0.5859	0.8868	0.046*	
H15E	0.2053	0.4294	0.9437	0.046*	
H15F	0.0639	0.5550	0.9871	0.046*	
C16A	−0.1904 (12)	0.4126 (9)	0.4946 (7)	0.036 (2)	
H16D	−0.1881	0.3525	0.4356	0.054*	
H16E	−0.1646	0.5117	0.4687	0.054*	
H16F	−0.3231	0.4056	0.5354	0.054*	
O2	0.4216 (8)	0.4614 (6)	0.7244 (5)	0.0338 (14)	
Br3	0.14860 (10)	0.09484 (7)	0.65374 (6)	0.0290 (2)	
Br4	−0.31319 (12)	0.12276 (9)	0.62628 (7)	0.0378 (3)	

### Atomic displacement parameters (Å^2^)

**Table:**

	*U*^11^	*U*^22^	*U*^33^	*U*^12^	*U*^13^	*U*^23^
C1	0.029 (5)	0.015 (4)	0.039 (5)	0.006 (3)	−0.006 (4)	−0.002 (3)
C2	0.032 (5)	0.042 (5)	0.027 (5)	0.003 (4)	−0.011 (4)	0.003 (4)
C3	0.028 (5)	0.038 (6)	0.021 (5)	0.002 (4)	−0.012 (4)	−0.006 (4)
C4	0.020 (4)	0.028 (4)	0.027 (4)	0.000 (3)	−0.003 (3)	0.005 (3)
C5	0.014 (4)	0.030 (4)	0.022 (4)	0.011 (3)	0.002 (3)	−0.002 (3)
C6	0.018 (4)	0.019 (4)	0.022 (4)	0.002 (3)	−0.008 (3)	−0.001 (3)
C7	0.020 (4)	0.021 (4)	0.025 (4)	0.005 (3)	0.004 (3)	−0.003 (3)
C8	0.030 (5)	0.027 (4)	0.025 (5)	0.008 (3)	0.002 (4)	−0.002 (3)
C9	0.029 (5)	0.030 (5)	0.042 (6)	−0.008 (4)	−0.015 (4)	−0.007 (4)
C10	0.036 (5)	0.021 (4)	0.041 (5)	0.005 (3)	−0.013 (4)	−0.004 (4)
C11	0.030 (4)	0.023 (5)	0.031 (5)	0.000 (3)	−0.004 (4)	0.004 (4)
C12	0.019 (4)	0.021 (4)	0.027 (4)	0.001 (3)	−0.003 (3)	−0.009 (3)
C13	0.040 (6)	0.037 (5)	0.043 (6)	0.006 (4)	−0.018 (5)	0.006 (4)
C14	0.048 (6)	0.046 (6)	0.018 (5)	0.012 (4)	0.003 (4)	−0.007 (4)
C15	0.044 (6)	0.034 (5)	0.017 (4)	0.015 (4)	−0.012 (4)	−0.009 (4)
C16	0.024 (5)	0.050 (6)	0.034 (5)	−0.008 (4)	0.007 (4)	−0.003 (4)
O1	0.017 (3)	0.034 (3)	0.059 (4)	0.005 (2)	−0.001 (3)	0.000 (3)
Br1	0.0291 (5)	0.0234 (5)	0.0468 (6)	0.0026 (3)	−0.0041 (4)	−0.0068 (4)
Br2	0.0272 (5)	0.0480 (6)	0.0380 (6)	0.0191 (4)	−0.0027 (4)	−0.0084 (5)
C1A	0.017 (4)	0.031 (4)	0.024 (4)	0.000 (3)	0.005 (3)	0.000 (3)
C2A	0.028 (4)	0.028 (4)	0.026 (5)	0.006 (3)	0.010 (4)	0.001 (3)
C3A	0.020 (4)	0.024 (5)	0.027 (5)	0.001 (3)	0.005 (4)	−0.003 (4)
C4A	0.015 (4)	0.022 (4)	0.035 (5)	0.000 (3)	−0.004 (3)	0.000 (3)
C5A	0.018 (4)	0.029 (4)	0.016 (4)	−0.002 (3)	−0.001 (3)	−0.006 (3)
C6A	0.018 (4)	0.014 (4)	0.024 (4)	0.005 (3)	0.004 (3)	0.002 (3)
C7A	0.024 (4)	0.016 (4)	0.023 (4)	0.004 (3)	−0.003 (3)	−0.006 (3)
C8A	0.034 (5)	0.023 (4)	0.013 (4)	0.003 (3)	−0.003 (3)	−0.002 (3)
C9A	0.021 (4)	0.030 (4)	0.027 (5)	0.008 (3)	0.011 (4)	−0.004 (4)
C10A	0.038 (5)	0.017 (4)	0.043 (5)	0.013 (3)	0.009 (4)	0.001 (4)
C11A	0.024 (4)	0.012 (4)	0.039 (5)	0.004 (3)	0.002 (4)	0.003 (3)
C12A	0.021 (4)	0.018 (4)	0.031 (5)	0.004 (3)	0.002 (3)	−0.006 (3)
C13A	0.034 (5)	0.026 (5)	0.045 (6)	−0.004 (4)	0.003 (4)	−0.001 (4)
C14A	0.029 (5)	0.024 (4)	0.041 (5)	0.005 (4)	0.006 (4)	0.004 (4)
C15A	0.045 (5)	0.024 (4)	0.025 (5)	0.003 (4)	−0.010 (4)	−0.005 (3)
C16A	0.027 (5)	0.051 (6)	0.030 (5)	0.002 (4)	−0.006 (4)	−0.003 (4)
O2	0.016 (3)	0.035 (3)	0.050 (4)	0.001 (2)	−0.003 (3)	−0.001 (3)
Br3	0.0281 (5)	0.0210 (5)	0.0387 (6)	0.0047 (3)	−0.0057 (4)	−0.0052 (4)
Br4	0.0263 (5)	0.0444 (6)	0.0443 (6)	−0.0121 (4)	−0.0082 (4)	−0.0074 (5)

### Geometric parameters (Å, º)

**Table:**

C1—C12	1.525 (11)	C1A—C13A	1.539 (11)
C1—C11	1.537 (11)	C1A—C11A	1.552 (10)
C1—C13	1.547 (11)	C1A—C12A	1.557 (10)
C1—C2	1.564 (11)	C1A—C2A	1.557 (11)
C2—C3	1.534 (12)	C2A—C3A	1.524 (11)
C2—H2A	0.9900	C2A—H2C	0.9900
C2—H2B	0.9900	C2A—H2D	0.9900
C3—C4	1.534 (10)	C3A—C4A	1.532 (9)
C3—H3A	0.9900	C3A—H3C	0.9900
C3—H3B	0.9900	C3A—H3D	0.9900
C4—C5	1.517 (10)	C4A—C5A	1.490 (10)
C4—C16	1.519 (10)	C4A—C16A	1.529 (11)
C4—C6	1.548 (10)	C4A—C6A	1.534 (10)
C5—C6	1.506 (11)	C5A—C6A	1.518 (10)
C5—Br1	1.907 (8)	C5A—Br4	1.921 (7)
C5—Br2	1.930 (7)	C5A—Br3	1.933 (8)
C6—C7	1.527 (9)	C6A—C7A	1.503 (10)
C6—H6	1.0000	C6A—H6A1	1.0000
C7—C12	1.522 (10)	C7A—C12A	1.523 (10)
C7—C8	1.591 (10)	C7A—C8A	1.575 (10)
C7—H7	1.0000	C7A—H7A1	1.0000
C8—C14	1.526 (10)	C8A—C14A	1.526 (10)
C8—C15	1.555 (10)	C8A—C9A	1.530 (10)
C8—C9	1.556 (11)	C8A—C15A	1.551 (10)
C9—C10	1.513 (11)	C9A—C10A	1.545 (11)
C9—H9A	0.9900	C9A—H9A1	0.9900
C9—H9B	0.9900	C9A—H9A2	0.9900
C10—C11	1.542 (11)	C10A—C11A	1.512 (11)
C10—H10A	0.9900	C10A—H10C	0.9900
C10—H10B	0.9900	C10A—H10D	0.9900
C11—H11A	0.9900	C11A—H11C	0.9900
C11—H11B	0.9900	C11A—H11D	0.9900
C12—O1	1.217 (9)	C12A—O2	1.212 (9)
C13—H13A	0.9800	C13A—H13D	0.9800
C13—H13B	0.9800	C13A—H13E	0.9800
C13—H13C	0.9800	C13A—H13F	0.9800
C14—H14A	0.9800	C14A—H14D	0.9800
C14—H14B	0.9800	C14A—H14E	0.9800
C14—H14C	0.9800	C14A—H14F	0.9800
C15—H15A	0.9800	C15A—H15D	0.9800
C15—H15B	0.9800	C15A—H15E	0.9800
C15—H15C	0.9800	C15A—H15F	0.9800
C16—H16A	0.9800	C16A—H16D	0.9800
C16—H16B	0.9800	C16A—H16E	0.9800
C16—H16C	0.9800	C16A—H16F	0.9800
			
C12—C1—C11	111.9 (7)	C13A—C1A—C11A	110.5 (7)
C12—C1—C13	108.5 (7)	C13A—C1A—C12A	108.2 (7)
C11—C1—C13	109.4 (6)	C11A—C1A—C12A	112.0 (6)
C12—C1—C2	108.1 (6)	C13A—C1A—C2A	109.6 (6)
C11—C1—C2	110.6 (7)	C11A—C1A—C2A	110.4 (7)
C13—C1—C2	108.3 (7)	C12A—C1A—C2A	106.0 (6)
C3—C2—C1	116.4 (7)	C3A—C2A—C1A	116.6 (6)
C3—C2—H2A	108.2	C3A—C2A—H2C	108.2
C1—C2—H2A	108.2	C1A—C2A—H2C	108.2
C3—C2—H2B	108.2	C3A—C2A—H2D	108.2
C1—C2—H2B	108.2	C1A—C2A—H2D	108.2
H2A—C2—H2B	107.3	H2C—C2A—H2D	107.3
C4—C3—C2	113.0 (7)	C2A—C3A—C4A	114.3 (6)
C4—C3—H3A	109.0	C2A—C3A—H3C	108.7
C2—C3—H3A	109.0	C4A—C3A—H3C	108.7
C4—C3—H3B	109.0	C2A—C3A—H3D	108.7
C2—C3—H3B	109.0	C4A—C3A—H3D	108.7
H3A—C3—H3B	107.8	H3C—C3A—H3D	107.6
C5—C4—C16	119.2 (7)	C5A—C4A—C16A	116.7 (6)
C5—C4—C3	118.8 (6)	C5A—C4A—C3A	120.5 (6)
C16—C4—C3	113.7 (7)	C16A—C4A—C3A	112.8 (7)
C5—C4—C6	58.9 (5)	C5A—C4A—C6A	60.2 (5)
C16—C4—C6	118.0 (7)	C16A—C4A—C6A	117.5 (7)
C3—C4—C6	117.7 (7)	C3A—C4A—C6A	119.6 (6)
C6—C5—C4	61.6 (5)	C4A—C5A—C6A	61.3 (5)
C6—C5—Br1	121.7 (5)	C4A—C5A—Br4	121.0 (5)
C4—C5—Br1	121.1 (5)	C6A—C5A—Br4	118.9 (5)
C6—C5—Br2	116.7 (6)	C4A—C5A—Br3	120.7 (5)
C4—C5—Br2	119.3 (5)	C6A—C5A—Br3	119.2 (5)
Br1—C5—Br2	109.6 (4)	Br4—C5A—Br3	109.0 (3)
C5—C6—C7	121.8 (7)	C7A—C6A—C5A	122.4 (6)
C5—C6—C4	59.5 (5)	C7A—C6A—C4A	124.3 (6)
C7—C6—C4	124.2 (6)	C5A—C6A—C4A	58.5 (5)
C5—C6—H6	113.6	C7A—C6A—H6A1	113.6
C7—C6—H6	113.6	C5A—C6A—H6A1	113.6
C4—C6—H6	113.6	C4A—C6A—H6A1	113.6
C12—C7—C6	116.5 (6)	C6A—C7A—C12A	114.9 (6)
C12—C7—C8	110.2 (6)	C6A—C7A—C8A	112.2 (6)
C6—C7—C8	110.8 (6)	C12A—C7A—C8A	110.7 (6)
C12—C7—H7	106.2	C6A—C7A—H7A1	106.1
C6—C7—H7	106.2	C12A—C7A—H7A1	106.1
C8—C7—H7	106.2	C8A—C7A—H7A1	106.1
C14—C8—C15	108.8 (7)	C14A—C8A—C9A	107.6 (6)
C14—C8—C9	110.2 (7)	C14A—C8A—C15A	107.6 (6)
C15—C8—C9	109.0 (7)	C9A—C8A—C15A	110.3 (6)
C14—C8—C7	109.3 (6)	C14A—C8A—C7A	108.5 (6)
C15—C8—C7	107.4 (6)	C9A—C8A—C7A	112.9 (6)
C9—C8—C7	112.0 (6)	C15A—C8A—C7A	109.7 (6)
C10—C9—C8	118.1 (7)	C8A—C9A—C10A	118.4 (7)
C10—C9—H9A	107.8	C8A—C9A—H9A1	107.7
C8—C9—H9A	107.8	C10A—C9A—H9A1	107.7
C10—C9—H9B	107.8	C8A—C9A—H9A2	107.7
C8—C9—H9B	107.8	C10A—C9A—H9A2	107.7
H9A—C9—H9B	107.1	H9A1—C9A—H9A2	107.1
C9—C10—C11	113.4 (7)	C11A—C10A—C9A	113.2 (7)
C9—C10—H10A	108.9	C11A—C10A—H10C	108.9
C11—C10—H10A	108.9	C9A—C10A—H10C	108.9
C9—C10—H10B	108.9	C11A—C10A—H10D	108.9
C11—C10—H10B	108.9	C9A—C10A—H10D	108.9
H10A—C10—H10B	107.7	H10C—C10A—H10D	107.7
C1—C11—C10	116.0 (7)	C10A—C11A—C1A	116.8 (7)
C1—C11—H11A	108.3	C10A—C11A—H11C	108.1
C10—C11—H11A	108.3	C1A—C11A—H11C	108.1
C1—C11—H11B	108.3	C10A—C11A—H11D	108.1
C10—C11—H11B	108.3	C1A—C11A—H11D	108.1
H11A—C11—H11B	107.4	H11C—C11A—H11D	107.3
O1—C12—C7	118.5 (7)	O2—C12A—C7A	120.0 (7)
O1—C12—C1	120.7 (7)	O2—C12A—C1A	119.0 (7)
C7—C12—C1	120.6 (6)	C7A—C12A—C1A	121.0 (6)
C1—C13—H13A	109.5	C1A—C13A—H13D	109.5
C1—C13—H13B	109.5	C1A—C13A—H13E	109.5
H13A—C13—H13B	109.5	H13D—C13A—H13E	109.5
C1—C13—H13C	109.5	C1A—C13A—H13F	109.5
H13A—C13—H13C	109.5	H13D—C13A—H13F	109.5
H13B—C13—H13C	109.5	H13E—C13A—H13F	109.5
C8—C14—H14A	109.5	C8A—C14A—H14D	109.5
C8—C14—H14B	109.5	C8A—C14A—H14E	109.5
H14A—C14—H14B	109.5	H14D—C14A—H14E	109.5
C8—C14—H14C	109.5	C8A—C14A—H14F	109.5
H14A—C14—H14C	109.5	H14D—C14A—H14F	109.5
H14B—C14—H14C	109.5	H14E—C14A—H14F	109.5
C8—C15—H15A	109.5	C8A—C15A—H15D	109.5
C8—C15—H15B	109.5	C8A—C15A—H15E	109.5
H15A—C15—H15B	109.5	H15D—C15A—H15E	109.5
C8—C15—H15C	109.5	C8A—C15A—H15F	109.5
H15A—C15—H15C	109.5	H15D—C15A—H15F	109.5
H15B—C15—H15C	109.5	H15E—C15A—H15F	109.5
C4—C16—H16A	109.5	C4A—C16A—H16D	109.5
C4—C16—H16B	109.5	C4A—C16A—H16E	109.5
H16A—C16—H16B	109.5	H16D—C16A—H16E	109.5
C4—C16—H16C	109.5	C4A—C16A—H16F	109.5
H16A—C16—H16C	109.5	H16D—C16A—H16F	109.5
H16B—C16—H16C	109.5	H16E—C16A—H16F	109.5
